# Targeted high throughput sequencing in hereditary ataxia and spastic paraplegia

**DOI:** 10.1371/journal.pone.0174667

**Published:** 2017-03-31

**Authors:** Zafar Iqbal, Siri L. Rydning, Iselin M. Wedding, Jeanette Koht, Lasse Pihlstrøm, Aina H. Rengmark, Sandra P. Henriksen, Chantal M. E. Tallaksen, Mathias Toft

**Affiliations:** 1 Department of Neurology, Oslo University Hospital, Oslo, Norway; 2 Institute of Clinical Medicine, Faculty of Medicine, University of Oslo, Oslo, Norway; 3 Department of Neurology, Drammen Hospital, Vestre Viken Hospital Trust, Drammen, Norway; Odense University Hospital, DENMARK

## Abstract

Hereditary ataxia and spastic paraplegia are heterogeneous monogenic neurodegenerative disorders. To date, a large number of individuals with such disorders remain undiagnosed. Here, we have assessed molecular diagnosis by gene panel sequencing in 105 early and late-onset hereditary ataxia and spastic paraplegia probands, in whom extensive previous investigations had failed to identify the genetic cause of disease. Pathogenic and likely-pathogenic variants were identified in 20 probands (19%) and variants of uncertain significance in ten probands (10%). Together these accounted for 30 probands (29%) and involved 18 different genes. Among several interesting findings, dominantly inherited *KIF1A* variants, p.(Val8Met) and p.(Ile27Thr) segregated in two independent families, both presenting with a pure spastic paraplegia phenotype. Two homozygous missense variants, p.(Gly4230Ser) and p.(Leu4221Val) were found in *SACS* in one consanguineous family, presenting with spastic ataxia and isolated cerebellar atrophy. The average disease duration in probands with pathogenic and likely-pathogenic variants was 31 years, ranging from 4 to 51 years. In conclusion, this study confirmed and expanded the clinical phenotypes associated with known disease genes. The results demonstrate that gene panel sequencing and similar sequencing approaches can serve as efficient diagnostic tools for different heterogeneous disorders. Early use of such strategies may help to reduce both costs and time of the diagnostic process.

## Introduction

The spinocerebellar degenerative disorders; hereditary ataxias (HA) and hereditary spastic paraplegias (HSP) are heterogeneous disorders causing progressive gait difficulties due to degeneration of the cerebellum, corticospinal tracts, brainstem, and/or spinal cord [1]. These disorders are relatively rare with an estimated total prevalence of 13.9/100,000 in southeast Norway [2]. HA is characterized by progressive limb and gait ataxia, loss of coordination and disturbances of speech and oculomotor control. HSP is characterized by progressive spasticity and weakness of the lower limbs, the weakness often being mild relative to the spasticity [1, 2]. Onset is reported at all ages, and all monogenic modes of inheritances—autosomal dominant, autosomal recessive, and X-linked—have been identified [3]. To date, pathogenic variants in more than 100 genes have been identified in spinocerebellar degenerative disorders [4–7]. Identifying molecular diagnoses in such genetically heterogeneous disorders is challenging. Usually multitier, expensive and time-consuming investigations are performed. Nevertheless, a large number of affected individuals remain without a molecular diagnosis.

With the progress in sequencing technologies, there are several methods available to screen hundreds or thousands of genes at once and possibly identify a molecular diagnosis in a shorter time period at lower costs. Gene panel sequencing (GPS) or targeted high throughput sequencing, whole-exome sequencing (WES), and whole-genome sequencing (WGS) methods are currently being used by researchers and diagnostic laboratories. These methods have different advantages related to quality and interpretation of data, management of ethical issues, and economic effectiveness. Besides other high throughput sequencing methods, GPS has been proven successful in several heterogeneous neurological disorders [8–10].

In the present study, we have evaluated the use of GPS in 105 clinically well-characterized probands affected with HA or HSP in whom previous extensive investigations had failed to identify a genetic cause. The study provides insights into the value of this diagnostic strategy and illustrates the diversity of genetic causes of spinocerebellar degenerative disorders.

## Methods

### Participants

**Fig 1:**
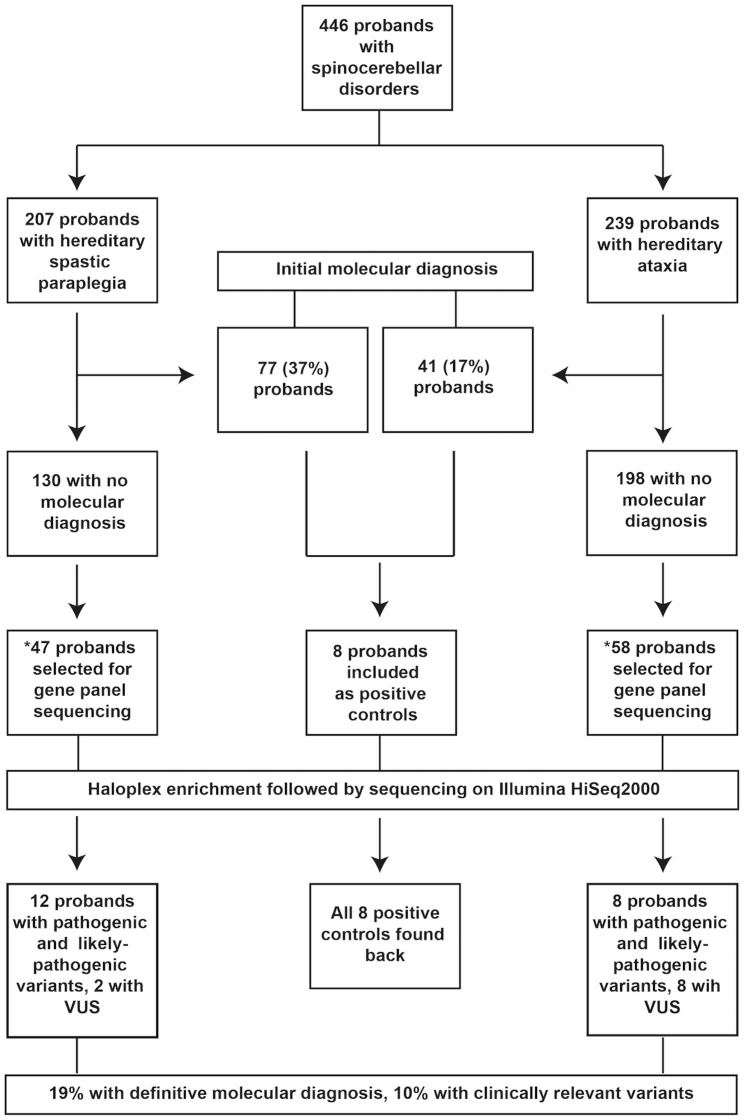
Clinical flowchart. The figure explains the selection of probands from the clinicogenetic database, and the resulting total number of molecular diagnoses. VUS: variants of uncertain significance. * Indicates selection criteria of 105 probands: 1) Verified family history; 2) Completed thorough investigations; 3) Availability of probands; 4) Sporadic cases considered to be HSP or HA, fulfilling 2) and 3).

In 2002, a research study was initiated at the Department of Neurology, Oslo University Hospital, carefully registering patients with HA and HSP in Norway. In 2014 the database consisted of 683 individuals with a diagnosis of HA and HSP, of whom 446 were probands [2]. The database has been designed to comprehensively cover the South-Eastern Norway health region where 55.8% of the Norwegian population lives. In addition, patients have been referred from the rest of the country since 2002. Main inclusion criteria for HA were cerebellar gait and/or limb ataxia, and for HSP, spasticity in the lower limbs, brisk reflexes and positive Babinski sign [11, 12]. In addition, most of the included probands had a known family history of disease. A minority had sporadic disease, which after thorough investigation was considered compatible with a hereditary type of spinocerebellar degenerative disorder. 17% of the HA probands and 37% of HSP probands had an exact genetic diagnosis (Fig 1) at start of the present study. Molecular investigations were carried out according to what was diagnostically available at the time of examinations. All HA probands were previously screened for SCA1, SCA2, SCA3, SCA6, SCA7, and for Friedreich ataxia in recessive and sporadic cases. HSP probands were screened for variants in the genes linked to SPG4, SPG3A, and most also for SPG31. To detect gene-dosage defects, multiple ligation-dependent probe amplification (MLPA) was performed in all HSP probands for SPG4 and SPG3A. Additional molecular tests were performed depending on the phenotype and the pedigree structure, including variants in the genes linked to; SPG7, SPG1, SPG2, FXTAS, POLG, SCA8, SPG11, AOA1, AOA2, Ataxia Telangiectasia, ARSACS, SPG8, DRPLA, and SPG42. Array comparative genomic hybridization (aCGH) was performed in all probands with cognitive impairment. Also, biochemical tests for metabolic disorders such as adrenoleukodystrophy and gangliosidosis, as well as biomarkers as carbohydrate-deficient transferrin, albumin, cholesterol, gamma globulins, alpha-fetoprotein and vitamin E were tested when relevant. Brain magnetic resonance imaging (MRI) was performed in most of the probands.

According to the protocol, 105 of the 328 probands without molecular diagnosis in the database could be selected for analysis in the study. They were selected from the database according to the following criteria: 1. Verified family history, 2. Completed thorough investigations, including screening for differential diagnoses and the above mentioned molecular analyses, and 3. Availability of probands. All probands (n = 89) fulfilling these three criteria were included. In addition,16 sporadic cases where other causes had been excluded, and HSP or HA remained the most likely diagnosis were included (see Fig 1, and Table 1). To validate our study design, we also included eight samples from the database with known pathogenic variants as positive controls (S1 Table). This project was approved by the Regional Committee for Medical and Health Research Ethics, southeast Norway under ethical agreement REK 2010/1579a. Written informed consent was obtained from all study participants.

**Table 1:** Clinical characteristics of the 105 probands included in the study.

Classification of probands	Total	AD	AR	Sporadic	Pure^a^	Complex^a^	Episodic^a^	Male/female	Childhood onset (<18 years)	Adult onset	Range of age at onset	Average age of onset^b^	Range of disease duration	Average disease duration^d^	Average disability stage^e^
HA	58	42	10	6	28	30	4	27/31	20	38	0–79 y^c^	35 y	2–72 y	22 y	3.4
HSP	47	26	11	10	19	28	0	25/22	21	26	1–64 y	26 y	2–53 y	21 y	3.4
Total (percentage)	105	68 (65%)	21 (20%)	16 (15%)	47 (45%)	58 (55%)	4 (3.8%)	52/53 (49.5%/50.5%)	41 (39%)	64 (61%)	0–79 y	31 y	2–72 y	21.7 y	3.4

Abbreviations: AD, Autosomal dominant; AR; autosomal recessive.

^a^Phenotypic classification; pure HSP or HA or complex disorder with additional symptoms.

^b^age at onset of clinical symptoms of the disease.

^c^number in years; y.

^d^Disease duration at the time of examination.

^e^Disability stage 1–6; 1:Signs at examination; 2:Mild, able to run; 3:Moderate, limited walking without aid; 4:Severe, walking with one stick, 5:Walking with two sticks; 6:Requiring wheelchair.

### Molecular genetics and bioinformatic analyses

The SureDesign tool (Agilent Technologies, Santa Clara, CA) was used to create a Haloplex custom gene panel targeting 159 genes (including the 10-bp flanking sequence on both sides of each exon) involved in different neurodegenerative disorders. The gene panel included 91 genes (S2 Table) reported to be definitively or possibly implicated in classical HA and HSP presentations at the time of study design (January 2014). Preparation of DNA pools from ten individuals was carried out as described before [13]. Target enrichment was performed according to the instructions of the Haloplex Target enrichment system for Illumina Sequencing Version D.5, May 2013. 100bp paired-end sequencing using a single lane on an Illumina HiSeq2000 instrument (Illumina, Santa Clara, CA) was performed at the Norwegian Sequencing Centre, Oslo. We also sequenced and analyzed 230 healthy controls using the same approach. The in-house bioinformatic pipeline has been described in details elsewhere [13]. For the variant filtering process, we considered only nonsense and missense variants, indels, and variants at canonical splice sites, excluding variants with minor allele frequency greater than 0.01 in different public and local resources; 1000g data (http://www.1000genomes.org), Exome Sequencing Project (ESP, http://evs.gs.washington.edu/EVS/), Exome Aggregation Consortium (ExAC, http://exac.broadinstitute.org) data, 176 ethnically-matched in-house exomes, and the 230 ethnically-matched internal controls. Moreover, we used the combined annotation dependent depletion (CADD) [14] tool to predict possible functional effects of a variant. We used a cut-off value of Phred-scaled CADD score >12, based on the value found for previously known pathogenic variants in our positive controls (S1 Table), as well as documented elsewhere [15]. The variants were examined by visual inspection of the sequence alignment/map format files to remove sequencing errors. Available non-affected and affected family members were tested for segregation of identified variants in the respective families. For any identified variant, all kinds of phenotypic presentations were considered in order to allow the clinical variability. After the initial filtering process, we followed the guidelines to interpret sequence variants provided by the joint consensus recommendations of the American College of Medical Genetics and Genomic (ACMG) and the Association for Molecular Pathology (AMP). This recommends the use of specific standard terminology to classify sequence variants into different classes; pathogenic, likely-pathogenic, and variants of uncertain significance (VUS) [16]. We will refer to these criteria as the ACMG criteria. All the presented variants have been submitted to the Leiden Open Variation Database (LOVD) server (http://databases.lovd.nl/shared), and any additional information on the sequencing data can be shared on request.

### Sanger sequencing

Variants identified by GPS were confirmed and validated by Sanger sequencing (S1 Appendix). In order to investigate the location of the variants in the genome, as well as to assign evolutionary conservation score (PhyloP) and functional predictions to the variants by several *in-silico* programs (Polyphen2, SIFT, and MutationTaster), Alamut 2.8.0v (http://alamut.interactive-biosoftware.com) was used.

## Results

### Clinical presentation

The clinical characteristics of the 58 HA and 47 HSP probands are described in Table 1. The inheritance pattern was presumed autosomal dominant (AD) in 68 (65%) and autosomal recessive (AR) in 21 (20%). Sixteen (15%) were sporadic (SPO) cases. Of the autosomal recessive probands, seven had consanguinity in the family history. The clinical phenotype was pure in 45% and complex in 55% of cases. Four HA probands presented with episodic ataxia. The average age of onset was 30.7 years, with a range from birth to 79 years of age. 39% of the probands had childhood onset of disease, with first symptoms starting before 18 years of age. Disease duration at inclusion in the database [2] was on average 22 years with a range from 2 to 72 years.

### Genetic analysis

High quality sequencing data was obtained with an average of 99% bases covered >80x in the targeted regions (S1 Fig). Our bioinformatic analyses identified 1182 variants, including single nucleotide variants and indels. All eight positive controls were identified in the data, confirming the sensitivity of the used method (S1 Table). By applying our filtering criteria and the ACMG guidelines for variant classification, we identified 20 probands (19%) carrying pathogenic and likely-pathogenic variants (Table 2). The allele frequencies of these variants in local and public databases are presented in S3 Table. Ten probands (10%) were identified with VUS (Table 3). Together these accounted for 30 probands (29%). Of these, 16 are from HA and 14 from HSP categories (Fig 1, Tables 2 and 3).

**Table 2:** Pathogenic and likely-pathogenic variants identified in hereditary ataxia and spastic paraplegia probands.

Chr	Gene	Disorder	OMIM phenotype#	Individual identity	Genomic position (Hg19/GRCh37)	Transcript	cDNA position	Aamino acid position	LOVD variant ID	Zygosity	PhyloP score, In-silico pathogenicity predictions, CADD	Allele frequency in ExAC	Number of affected individuals carrying the variant in the respective family	ACMG classification. In case of known pathogenic variant, HGMD accession#
1	*KCND3*	SCA19 (AD)	607346	HCT-095	g.112329705G>A	NM_004980.4	c.1130C>T	p.(Thr377Met)	162972	het	5.77/s,m,p/26.3	-	1^#^	P, CM1212997
2	*SPAST*	SPG4 (AD)	182601	HCT-020	g.32341274G>C	NM_014946.3	c.1091G>C	p.(Arg364Thr)	162973	het	4.73/s,m,p/21	-	2	P, CM076534
2	*KIF1A*	SPG30 (AD)	614255	HCT-024	g.241737090A>G	NM_001244008.1	c.80T>C	p.(Ile27Thr)	162974	het	4.64/s,m,p/24.3	-	6	LP
2	*KIF1A*	SPG30 (AD)	614255	HCT-026	g.241737148C>T	NM_001244008.1	c.22G>A	p.(Val8Met)	162975	het	5.69/s,m,p/29.8	-	3	LP
2	*REEP1*	SPG31 (AD)	610250	HCT-018	g.86481833A>G	NM_001164730.1	c.308T>C	p.(Leu103Pro)	162976	het	5.05/s,m,p/19.2	-	1	LP
2	*REEP1*	SPG31 (AD)	610250	HCT-049	g.86491145C>T	NM_001164730.1	c.146G>A	p.(Trp49*)	162977	het	NA/NA/NA	-	1^#^	P
3	*ITPR1*	SCA15/29 (AD)	606658/117360	HCT-080	g.4776923A>T	NM_001168272.1	c.5384A>T	p.(Glu1795Val)	162978	het	4.89/s,m/19.9	-	4	LP
11	*BSCL2*	SPG17 (AD)	270685	HCT-051	g.62469965G>A	NM_001122955.3	c.461C>T	p.(Ser154Leu)	162979	het	4.97/s,m,p/29.4	-	1^#^	P, CM040382
11	*SPTBN2*^a^	SCA5 (AD)	600224	HCT-102	g.66472866_66472868del	NM_00694.2	c.1879_1881del	p.(Cys627del)	162980	het	NA/NA/NA	-	2	LP
12	*KIF5A*	SPG10 (AD)	604187	HCT-043	g.57962782G>A	NM_004984.2	c.751G>A	p.(Glu251Lys)	162981	het	5.29/s,m,p/33	-	4	P, CM090637
13	*SACS*	ARSACS (AR)	270550	HCT-106	g.23905327C>T	NM_014363.4	c.12688G>A	p.(Gly4230Ser)	162982	hom	6.02/s,p/27.3	0.000008276	1	LP
13	*SACS*	ARSACS (AR)	270550	HCT-106	g.23905354G>C	NM_014363.4	c.12661C>G	p.(Leu4221Val)	162983	hom	2.47/s,p/14.7	-	1	LP
14	*ATL1*	SPG3A (AD)	182600	HCT-025	g.51088610T>C	NM_015915.4	c.1040T>C	p.(Met347Thr)	162984	het	4.89/s,m,p/21.2	-	2	P, CM111079
16	*SPG7*	SPG7 (AR)	607259	HCT-048	g.89576947T>A	NM_003119.2	c.233T>A	p.(Leu78*)	162985	hom	NA/NA/NA	0.0004725	1^#^	P, CM081826
16	*SPG7*	SPG7 (AR)	607259	HCT-033	1. g.89613145C>T 2. g.89616910A>T	NM_003119.2	1. c.1529C>T, 2. c.1672A>T	1. p.(Ala510Val) 2. p.(Lys558*)	1. 162986 2. 162987	c.het	1. 6.02/s,m,p/26.1 2. NA/NA/NA	1. 0.002522 2. 0.0001403	2	1. P, CM085726; 2. P, CM129285
16	*SPG7*	SPG7 (AR)	607259	HCT-112	1. g.89613145C>T 2. g.89620367A>C	NM_003119.2	1. c.1529C>T, 2. c.2102A>C	1. p.(Ala510Val) 2. p.(His701Pro)	1. 162986 2. 162988	c.het	1. 6.02/s,m,p/26.1 2. 2.22/m/12.8	1. 0.002522 2. 0.00003399	2	1. P, CM085726; 2. P, CM164152
16	*SPG7*	SPG7 (AR)	607259	HCT-116	1. g.89613145C>T 2. g.89620367A>C	NM_003119.2	1. c.1529C>T, 2. c.2102A>C	1. p.(Ala510Val) 2. p.(His701Pro)	1. 162986 2. 162988	c.het	1. 6.02/s,m,p/26.1 2. 2.22/m/12.8	1. 0.002522 2. 0.00003399	2	1. P, CM085726; 2. P, CM164152
18	*AFG3L2*^b^	SCA28 (AD)	610246	HCT-067	g.12337401A>G	NM_006796.2	c.2114T>C	p.(Ile705Thr)	162989	het	4.73/s,m,p/22.9	-	2	LP
19	*CACNA1A*	EA2 (AD)	108500	HCT-059	g.13323200G>A	NM_001127222.1	c.6187C>T	p.(Gln2063*)	162990	het	NA/NA/NA	-	3	P
19	*PRKCG*	SCA14 (AD)	605361	HCT-118	g.54393142T>C	NM_002739.3	c.400T>C	p.(Cys134Arg)	162991	het	2.87/m,p/18.7	-	1^#^	LP
20	*TGM6*^c^	SCA35 (AD)	613908	HCT-101	g.2384113G>A	NM_198994.2	c.1060G>A	p.(Val354Ile)	162992	het	3.68/s,m,p/21.2	-	1^#^	LP

Abbreviations: Chr, chromosome; AD, autosomal dominant; AR, autosomal recessive; OMIM, online Mendelian inheritance in man; cDNA, complementary deoxyribonucleic acid; Zygosity, heterozygous (het), compound heterozygous (c.het), homozygous (hom); LOVD, Leiden open variation database; CADD, combined annotation dependent depletion score, also called as a PHRED score; ExAC, exome aggregation consortium (http://exac.broadinstitute.org); HGMD, human gene mutation database; ACMG, American college of medical genetics; PhyloP, evolutionary conservation score at specific nucleotide position; s, damaging prediction by SIFT (http://sift.jcvi.org); m, damaging prediction by MutationTaster (http://www.mutationtaster.org); p, damaging prediction by PolyPhen-2 (http://genetics.bwh.harvard.edu/pph2/); P, pathogenic; LP, likely-pathogenic; NA,not available or not applicable. OMIM gene identifiers: *KCND3* (605411), *SPAST* (604277), *KIF1A* (601255), *REEP1* (609139), *ITPR1* (147265), *BSCL2* (606158), *SPTBN2* (604985), *KIF5A* (602821), *SACS* (604490), *ATL1* (606439), *BEAN1* (612051), *SPG7* (602783), *AFG3L2* (604581), *CACNA1A* (601011), *PRKCG* (176980), *RTN2* (603183), TGM6 (613900).

^a^3q26 duplication was previously found in the same family, indicating two independent genetic mutations.

^b^*SPG7* variant, c.C1529T, p.(Ala510Val) was heterozygously present in individual HCT-067.

^c^Another variant in a recessive gene *ZFYVE26* was found heterozygously, c.7055C>T, p.(Thr2352Ile).

^#^, No additional samples of affected and/or unaffected individuals were available for segregation analysis.

**Table 3:** List of variants of uncertain significance.

Chr	Gene	Disorder	OMIM phenotype#	Individual identity	Genomic position (Hg19/GRCH 37)	Transcript	cDNA position	Amino acid position	LOVD variant ID	Zygosity	PhyloP score, in-silico pathogenicity predictions, CADD	Allele frequency in ExAC	Number of affected individuals carrying the variant in the respective family	Main phenotype—additional features
1	*KCND3* ^a^	SCA19 (AD)	607346	HCT-088	g.112322852T>C	NM_004980.4	c.1456A>G	p.(Thr486Ala)	162993	het	4.40/m,p/19.2	0.001461	1	comp AT—pyramidal and extrapyramidal signs
3	*ITPR1*	SCA15/29 (AD)	606658/117360	HCT-029	g.4735396G>A	NM_001168272.1	c.4207G>A	p.(Val1403Met)	162994	het	4.08/s,m,p/17.1	0.00004663	1	pure HSP—none
3	*ITPR1* ^b^	SCA15/29 (AD)	606658/117360	HCT-077	g.4810224G>A	NM_001168272.1	c.5710G>A	p.(Glu1904Lys)	162995	het	3.68/s,m/13.3	0.000008432	1^#^	comp AT—early onset, spastic AT
11	*BSCL2*	SPG17 (AD)	270685	HCT-044	g.62462158C>A	NM_001122955.3	c.512G>T	p.(Arg171Leu)	162996	het	2.14/s,m,p/19.3	0.000008322	1	pure HSP—amyotrophy, neuropathy
11	*SPTBN2*	SCA5 (AD)	600224	HCT-086	g.66453485T>G	NM_00694.2	c.7030A>C	p.(Ser2344Arg)	162997	het	1.66/p/15.1	0.00001679	1^#^	comp AT—neuropathy
11	*SPTBN2*^c^	SCA5 (AD)	600224	HCT-071	g.66453406C>T	NM_00694.2	c.7109G>A	p.(Arg2370His)	162998	het	5.86/s,m,p/33	0.0001252	1^#^	pure AT—none
12	*KIF5A*	SPG10 (AD)	604187	HCT-082	g. 57970109C>T	NM_004984.2	c.2146C>T	p.(Arg716Trp)	162999	het	3.60/s,m,p/24.6	0.00005826	1^#^	comp AT—episodic
15	*TTBK2*	SCA11 (AD)	604432	HCT-115	g.43132604C>G	NM_173500.3	c.245G>C	p.(Gly82Ala)	163000	het	5.21/s,m,p/16	0.0002898	1	comp AT—spastic AT
16	*BEAN1*	SCA31 (AD)	117210	HCT-087	g.66503607T>A	NM_001178020.2	c.128T>A	p.(Ile43Lys)	163001	het	3.35/s,m,p/25.3	-	2	comp AT—lower limb paresis, neuropathy
19	*RTN2*	SPG12 (AD)	604805	HCT-057	g.45996535C>A	NM_005619.3	c.916G>T	p.(Val306Phe)	163002	het	2.71/p/15.6	-	3	pure AT—none

Abbreviations: Chr, chromosome; AD, autosomal dominant; AR, autosomal recessive; OMIM, online Mendelian inheritance in man; cDNA, complementary deoxyribonucleic acid; Zygosity, heterozygous (het), compound heterozygous (c.het), homozygous (hom); LOVD, Leiden open variation database; CADD, combined annotation dependent depletion score, also called as a PHRED score PhyloP, evolutionary conservation score at specific nucleotide position; s, damaging prediction by SIFT (http://sift.jcvi.org); m, damaging prediction by MutationTaster (http://www.mutationtaster.org); p, damaging prediction by PolyPhen-2 (http://genetics.bwh.harvard.edu/pph2/); ExAC, exome aggregation consortium (http://exac.broadinstitute.org); comp, complex; AT, ataxia; HSP, hereditary spastic paraplegia. OMIM gene identifiers: *KCND3* (605411), *ITPR1* (147265), *BSCL2* (606158), *SPTBN2* (604985), *KIF5A* (602821), *TTBK2* (611695).

#### Pathogenic and likely-pathogenic variants

In total, pathogenic and likely-pathogenic variants were found in 15 genes. Identified variants in the genes *KCND3-*p.(Thr377Met), *SPAST*-p.(Arg364Thr), *BSCL2-*p.(Ser154Leu), *KIF5A-*p.(Glu251Lys), *ATL1-*p.(Met347Thr), and *SPG7-*p.(Leu78*), p.(Ala510Val), p.(Lys558*), and p.(His701Pro) have already been reported in the literature as pathogenic [17–24], while the rest of the variants found are categorized as novel pathogenic or likely-pathogenic variants (Table 2). Of the pathogenic and likely-pathogenic variant carriers, 12 probands belonged to the childhood-onset category (<18 years), and eight had adult-onset, resulting in a diagnostic yield of 29% and 12.5% in the respective categories. The average disease duration in probands with identified pathogenic and likely-pathogenic variants was 31 years (range 4–51 years) (Table 4). Diagnostic rates for different categories such as AD, AR, SPO, consanguinity, pure, and complex forms of the disease are presented in S4 Table. In all the 20 families with identified pathogenic or likely-pathogenic variants, the clinical symptoms and findings were concordant with previously published descriptions of the respective corresponding disorders. The phenotypic details of these 20 probands are documented in Table 4 as well as in S2 Appendix.

**Table 4:** Clinical features of the probands with definitive molecular diagnosis having pathogenic and likely-pathogenic variants.

No.	Individual	Gene/disorder Phenotype	Inheritance	Sex	Phenotype	Age at onset	Age at exam	Disease duration	Disability stage	First symptom	Spasticity***	Other findings	Sensory deficit****	Dysarthria****	Cognitive impairment	Ophthalmological findings	MRI	EMG/ENG
1	HCT-095	*KCND3*/SCA19	AD	F	pure AT	18	48	30	3	unsteadiness	0		0	0	0	saccadic pursuit	vermis atrophy	NA
2	HCT-020	*SAPST*/SPG4	AD	M	pure HSP	55	68	13	4	unsteadiness	LL		0	0	0	0	NA	NA
3	HCT-024	*KIF1A*/SPG30	AD	F	pure HSP	12	47	35	3	spasticity	LL		0	0	0	0	NA	normal
4	HCT-026	*KIF1A*/SPG30	AD	M	pure HSP	10	61	51	3	spasticity	LL		0	0	0	0	NA	NA
5	HCT-018	*REEP1*/SPG31	AD	F	comp HSP	20	67	47	2	unsteadiness	LL	distal amyotrophy	1	0	0	0	normal	NA
6	HCT-049	*REEP1*/SPG31	AD	M	pure HSP	4	36	32	4	spasticity	LL		0	0	NA	0	normal	axonal sensory
7	HCT-080	*ITPR1*/SCA15/29	AD	M	comp AT	10	51	41	4	unsteadiness	0	motor deficit	0	1	0	saccadic pursuit	NA	NA
8	HCT-051	*BSCL2*/SPG17	SPO	M	pure HSP	14	29	15	2	spasticity	LL		0	0	0	0		axonal and demyelinating sensorimotor
9	HCT-102	*SPTBN2*/SCA5	AD	M	pure AT	15	63	48	3	unsteadiness	0	pain (legs)	1	1	0	saccadic poursuit, OPN **, nystagmus	cerebellar atrophy	axonal sensorimotor
10	HCT-043*	*KIF5A*/SPG10	AD	F	pure HSP	5	39	34	3	shuffling	UL, LL	pain (legs)		1	1		normal	axonal sensorimotor
11	HCT-106	*SACS*/ARSACS	AR	M	comp AT	15	34	19	3	clumsiness	0	0	0	1	0	saccadic pursuit	cerebellar atrophy	axonal sensorimotor
12	HCT-025	*ATL1*/SPG3A	AD	F	comp HSP	1	32	31	6	unsteadiness	LL	pain (pelvis),motor deficit, tremor	1	0	0	0	NA	NA
13	HCT-048	*SPG7*/SPG7	AR	M	comp HSP	40	44	4	3	stiff legs	LL	motor deficit, extremity ataxia	1	0	0	saccadic pursuit	cerebellar atrophy	normal
14	HCT-033	*SPG7*/SPG7	AR	M	comp HSP	30	45	15	5	spasticity	LL	motor deficit	0	0	1	NA	cerebellar atrophy	normal
15	HCT-112	*SPG7*/SPG7	AR	M	comp HSP	14	62	48	5	unsteadiness	LL	motor deficit	1	1	0	saccadic pursuit, OPN **	cerebellar atrophy	NA
16	HCT-116	*SPG7*/SPG7	AR	F	comp HSP	20	43	23	3	spasticity and unsteadiness	LL	amyotrophy and motor deficit	0	0	0	saccadic pursuit	cerebellar atrophy	NA
17	HCT-067	*AFG3L2*/SCA28	AD	M	pure AT	20	48	28	3	unsteadiness	0	0	0	1	0	saccadic pursuit	cerebellar atrophy	normal
18	HCT-059	*CACNA1A*/EA2	AD	M	comp AT	1	42	41	1	episodic	0	decreased reflexes	0	0	0	nystagmus, saccadic pursuit	NA	NA
19	HCT-118	*PRKCG*/SCA14	AD	F	comp AT	23	48	25	2	unsteadiness	LL	joint pain	0	1	0	saccadic pursuit	cerebellar atrophy	NA
20	HCT-101	*TGM6*/SCA35	SPO	M	comp AT	10	55	45	4	clumsiness	LL	distal amyotrophy	0	0	0	saccadic pursuit	NA	NA

Abbreviations: AT, ataxia; HSP, hereditary spastic paraplegia; comp, complicated; AD, autosomal dominant; AR, autosomal recessive; SPO, sporadic; MRI, magnetic resonance imaging; EMG/ENG, electromyography/electroneurography.

* Also: dup 22q11.21.dup,

** ophthalmoplegia,

*** 0: no spasticity, UL: upper limb spasticity, LL: lower limb spasticity. NA: not available.

****0: Not present, 1:Present.

#### SPG30

**Fig 2:**
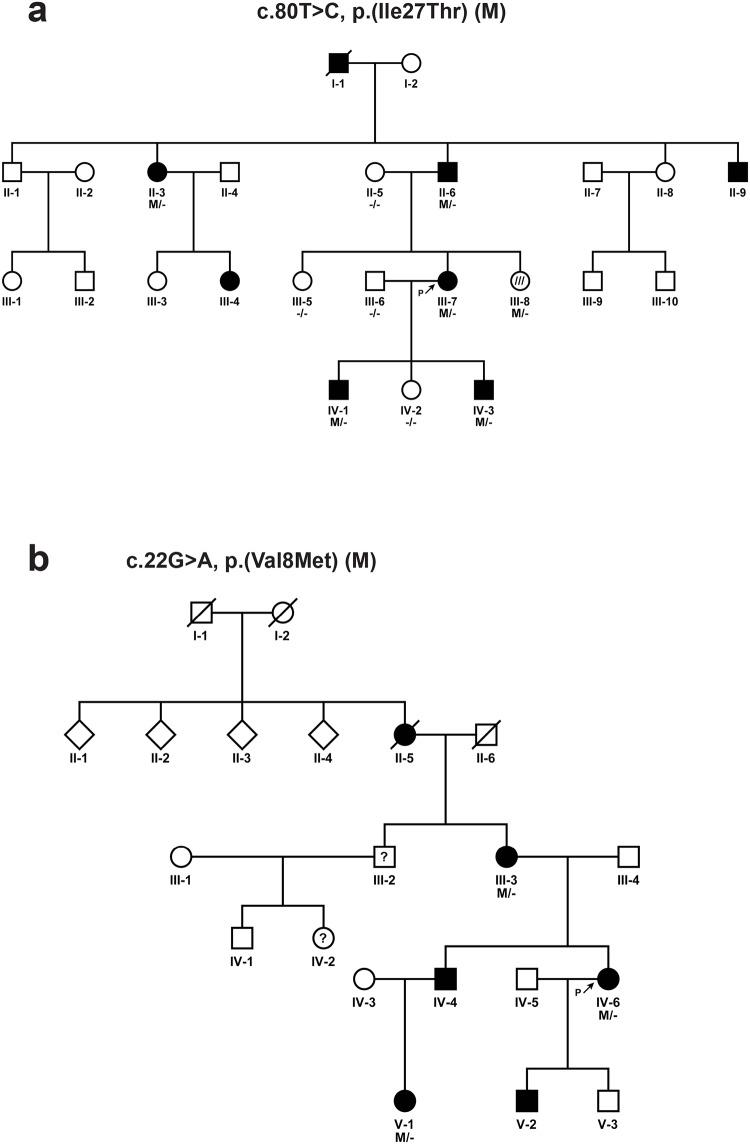
Pedigree structures of families with *KIF1A* variants. (a) Pedigree structure of family HCT-024 (III-7) with a c.80T>C, p.(Ile27Thr) variant in *KIF1A*. The filled symbols indicate affected individuals. The striped symbol indicates an individual that was initially classified as a non-affected individual, but after clinical re-examination was also found to be possibly affected. (b) Pedigree structure of family of HCT-026 (IV-6) with a c.22G>A, p.(Val8Met) variant. The symbols with a question mark are not confirmed regarding the phenotype. The diamond shaped symbols indicate masked gender. A line crossing a symbol represents a deceased individual. Probands are labelled with ‘P’.

Two novel variants in the *KIF1A* gene (SPG30, MIM 610357), p.(Ile27Thr) in probands HCT-024 (III-7) and p.(Val8Met), in HCT-026 (IV-6) were identified. Both variants segregated with the phenotype in these families with an autosomal dominant inheritance pattern (Table 2; Fig 2a and 2b). In the family of proband HCT-024 there were eight affected individuals in four successive generations (Fig 2a). DNA samples were available from five affected individuals with a pure HSP phenotype for segregation analysis, which revealed that all five carried the variant. Five individuals without subjective symptoms were also tested, of which one (III-8) carried the variant (Fig 2a). At the age of 31 years this subject had increased reflexes in the lower limbs. This was interpreted as a possible sign of disease, but extensor plantar reflex was not observed. Both families with *KIF1A* variants presented with a childhood onset, slowly progressive spastic paraplegia (Table 4). None of the affected individuals in these families had signs of cognitive impairment, ataxia or neuropathy, which may be present in complex HSP phenotypes.

#### ARSACS

**Fig 3:**
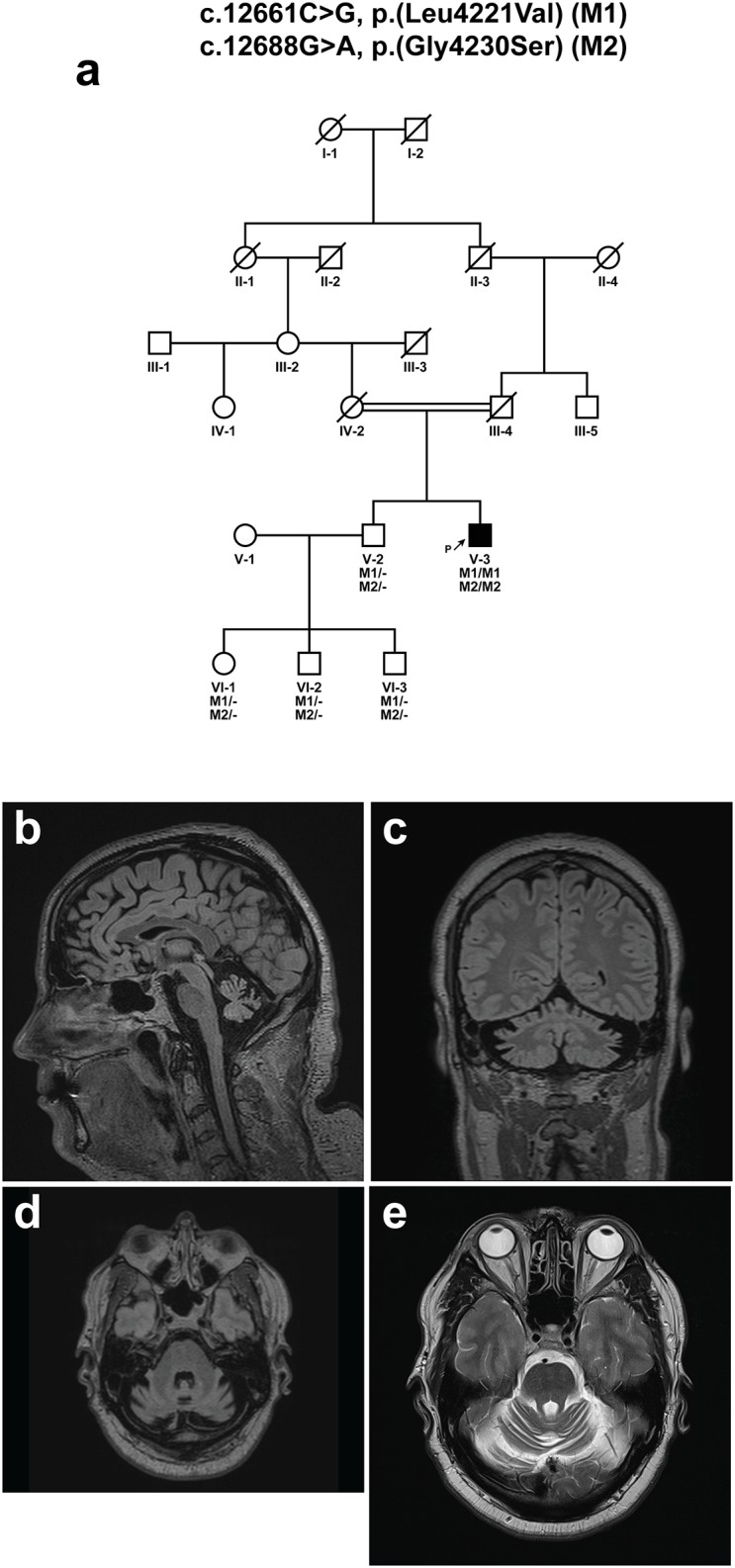
Pedigree structure and MRI scans of a family with *SACS* variants. (a) Pedigree structure of family HCT-106 (V-3) with a c.12688G>A, p.(Gly4230Ser) and c.12661C>G, p.(Leu4221Val) variants in *SACS*. A consanguineous marriage between individuals IV-2 and III-4 is indicated by a double line. Cerebral MRIs of HCT-106 at disease duration of 28 years in (b) FLAIR sequence in midline sagittal plane, (c) FLAIR sequence in coronal plane at the level of dorsal aspect of cerebellum, (d) FLAIR sequence in transversal plane at the level of the middle cerebellar peduncles, and (e) T2 sequence in transversal plane at the level of the superior cerebellar peduncles, showing atrophy of the cerebellar hemispheres and vermis with widening of fissures and folia.

Two novel homozygous variants, p.(Gly4230Ser) and p.(Leu4221Val) in the *SACS* gene were identified in proband HCT-106 (V-3), presenting an autosomal recessive SACS (ARSACS, MIM 270550) phenotype (Fig 3a, Table 4). There was consanguinity in this ethnic Norwegian family, and both variants were homozygously present in the only affected member of the family (Fig 3a). It is difficult to determine which variant is causing the disease, or whether both are involved. Both variants are extremely rare and were predicted to possibly affect protein function, although the evidence is stronger for the p.(Gly4230Ser) variant by several *in-silico* predictions (Table 2). The proband HCT-106 experienced slowly progressive clumsiness, and unsteadiness from 15 years of age. Brain MRI at ages of 37 and 44 years revealed general cerebellar atrophy with no signs of pontine linear hypointensities, as well as normal cervical cord and corpus callosum (Fig 3b, 3c, 3d and 3e). No retinal changes were found by fundoscopy or optical coherence tomography (Table 4).

#### Variants of uncertain significance

Furthermore, we identified ten VUS in eight genes (Table 3). In five of the ten probands with VUS, the phenotype was considered to be concordant with previous descriptions of the respective disorders; HCT-044 (*BSCL2*_SPG17, MIM 270685), HCT-088 (*KCND3_*SCA19, MIM 607346), HCT-086 & HCT-071 (*SPTBN2*_SCA5, MIM 600224), and HCT-115 (*TTBK2*_SCA11, MIM 604432) (Table 3). The phenotypic details of all VUS are described in S2 Appendix. Eight of these ten variants were found with a very low allele frequency in ExAC, including the five variants with concordant phenotypes. Variants located in *BEAN1*, *RTN2*, and *TTBK2* are categorized under this category—mainly because the disease mechanism due to the missense variants has not been previously either established or well-consolidated in these genes (Table 3). Further independent reports and/or functional studies are warranted to establish whether these VUS could be relevant to the disease in these probands.

## Discussion

The brain is the most complex and sophisticated organ in our body. 84% of the human genes are expressed in the brain [25]. A small perturbation in the expression of genes in the brain could lead to serious consequences and a number of neurological disorders including HA and HSP. Today, routine investigation of these disorders often involves a large number of serial independent molecular tests after the clinical diagnosis has been made. Certain mutations are very common in some populations, thus narrowing down the required number of tests. Other populations show high numbers of rare genotypes, as so far seen in the Norwegian ataxia population [2]. A correct molecular diagnosis is important for affected individuals, providing certainty, preventing unnecessary diagnostic tests and giving access to relevant supportive therapies and genetic counseling.

By using high throughput sequencing methods, the time from disease onset to the identification of molecular diagnoses may be substantially reduced. In the probands that were diagnosed in this study, there was notable average disease duration of 31 years. Our results therefore confirm that GPS based diagnostics or similar sequencing methods should be used earlier in the diagnostic process. However, trinucleotide expansion disorders (SCA1,2,3,6,7 and Friedreich ataxia) are relatively frequent in most HA cohorts, and such expansions are generally not detectable by high throughput sequencing techniques [26, 27]. As suggested in guidelines, the most frequent trinucleotide expansions should be tested initially in HA [28], and if negative GPS and similar methods may be considered as the next level of investigation.

GPS has some advantages compared to WES and WGS. Firstly, this method provides high-quality sequencing data with excellent coverage of the selected genes. This means that the method can reliably identify variants. Previous studies using WES and WGS have demonstrated that a considerable proportion of coding regions of genes harboring disease-related variants are not covered [29–31]. Secondly, GPS can limit the genetic incidental findings that can raise issues of ethical approval and communication of the findings to the affected individuals or guardians. Recently, Neveling *et al* [32] reported that 10% of the families did not provide consent for DNA testing during pre-counseling because of the risk of incidental findings. On the other hand, pre- and post-counseling can be conveniently offered to the small minority of probands or families concerned about the incidental findings after WES or WGS analysis. However, there are guidelines and recommendations available on how to report incidental findings [33].

This study revealed a definitive molecular diagnosis in 19% of probands, a sizeable yield, particularly taking into account that this cohort was previously extensively investigated by a series of molecular and biochemical analyses. Previous studies have revealed a variable scale of diagnostic power. According to one study, 18% molecular diagnosis was achieved by studying 50 childhood and adult-onset HA probands with GPS [34]. In another study, a diagnostic yield of 25% was attained by GPS in SPG4-negative HSP cases [35]. A diagnostic yield of 21% was achieved by WES in a cohort of sporadic and familial HA cases [36]. Pyle *et al* [37] presented 64% diagnosis by WES in a mixed cohort of HA, although the number of probands (n = 22) screened was very low. Kara *et al* [38] performed a combination of Sanger and clinical exome sequencing in a cohort of complex HSP cases and found plausible genetic defects in 49% with overwhelming majority (31%) of SPG11 cases. Another clinical exome sequencing study in a cohort of HSP and HA revealed 22–34% range of diagnostic yield [39]. The clinical characteristics of the studied cohort can affect the variable diagnostic yield found in different studies. This is demonstrated by the higher diagnostic yield seen in childhood-onset cases (29%) as compared to adult-onset (12.5%) in our study, as is also seen in previous studies [40, 41]. However, our study cohort consisted of previously extensively diagnosed probands, which introduces a selection bias compared to naive patient populations.

A large number of cases remained unsolved. There are several possible reasons that could contribute to this. Firstly; a subset of probands might have been explained by causal variant in novel HA/HSP genes that are yet to be identified or were found during the study period. Such newly identified genes can be added into gene panels on a regular basis. Secondly; some disease-causing variants might be localized to the non-coding part of DNA. Thirdly; somatic variants, also including mosaicism could be the cause in some of the individuals. Fourthly; coding variants might have been missed due to problems related to target capture, sequencing, bioinformatic analyses or our data filtering strategy. The DNA pooling strategy used in our study might have caused a reduced sensitivity to identify certain variants, although our present studies have found high sensitivity of our protocol [13]. In general, current high throughput sequencing technologies are less efficient for identification of indels as well as large-scale copy number variations (CNV) than single nucleotide variants, and our chosen study design has limitations in this regard. Of note, in one of the probands in our study, a parallel WES study has identified an in-frame deletion in *SPTBN2* that was not detected by our bioinformatic analyses, but was witnessed upon direct inspection of aligned reads. On the other hand, we identified a molecular diagnosis in two probands HCT-020 (SPG4, *SPAST*) and HCT-049 (SPG31, *REEP1*) where the pathogenic variant was not identified by previous conventional single gene sequencing, further highlighting the quality and comprehensiveness of the method used here.

Our bioinformatic analysis was unbiased in the sense that we looked for variants independent of known inheritance patterns. This leads to some interesting findings, further expanding and/or confirming the clinical and genetic heterogeneity and phenotypic spectrum for certain entities. The *KIF1A* gene was initially reported in autosomal recessive HSP (SPG30) [42]. However, recently several independent reports have identified variants in this gene in autosomal dominant forms of HSP (MRD9, MIM 614255). Twenty-two probands with *de novo* variants are reported with complicated form of HSP including a recent case of PEHO syndrome (MIM 260565) [43]. However, a pure HSP phenotype has previously been presented in one family, with a dominantly segregating variant, p.(Ser69Leu) [44]. In a most recent study, two additional segregating dominantly inherited variants, p.(Tyr74Cys) and p.(Gln632*) have been identified [39]. In our study, we have identified two dominantly segregating *KIF1A* variants, p.(Val8Met) and p.(Ile27Thr), in two independent families. This further confirms the dominant mode of inheritance and allelic heterogeneity associated with *KIF1A*. Both variants are located within the functional motor domain of the KIF1A protein. Interestingly, affected individuals of both of our families presented with pure HSP with a childhood onset of the disease, concordant with the reported families in which dominant inherited variant was found. Based on these findings, we suggest testing *KIF1A* in HSP regardless of the phenotypic variability and inheritance pattern.

We identified one proband with two homozygous missense variants, p.(Gly4230Ser) and p.(Leu4221Val) in *SACS* with a relatively slowly progressive recessive spastic ataxia with onset in the teens. The phenotype was consistent with the mild ARSACS phenotype often seen in non-Quebec-born individuals, with late-onset and absence of the characteristic retinal findings described in Quebec-born ARSACS individuals. Radiologically, the findings were stable over the last seven years with cerebellar atrophy. Remarkably, the brain MRI showed no signs of the previously described characteristic features of ARSACS [45]. This demonstrates that the clinical course was not sufficient for diagnosis, and systematic unbiased methods such as GPS could identify atypical or previously unreported phenotypes.

We have found ten variants that are categorized as VUS. Some uncertainty regarding the involvement of these variants in disease will remain until further individuals are reported from other studies and/or specific functional data from *in-vitro* or *in-vivo* studies become available.

It is well-established that HSPs and HAs often overlap, both clinically and genetically. While performing molecular diagnosis, the choice of gene panel for these disorders is critical. In most of the contemporary GPS or clinical exome studies, the gene panel selection has been variable. Our gene panel covered a broad range of genes—known to be involved in spinocerebellar degenerative disorders at the time of study. By developing a broad gene panel, one can avoid spending additional costs and time on single gene analyses or different limited/sub gene panels that are usually commercially available. Overall, because of the recent advancement in sequencing technologies, cost is less of an issue when it comes to broad gene panels or clinical exome sequencing/WES, as several parallel cheap and efficient sequencing methods are available today. Conversely, repeated update of the gene panels can increase the total costs as compared to WES, which is a downside of the GPS. Moreover, in case of clinical exome sequencing, with an updated ethical approval the WES data can be re-analyzed later to further explore novel genes responsible for disease in undiagnosed cases: this cannot be done with GPS and is an obvious limitation of the GPS method.

In conclusion, GPS and similar sequencing methods are effective choices for diagnostic procedures in order to reduce the duration to obtaining a correct molecular diagnosis. To date, these procedures are not available or implemented in most clinics in the world, and consequently many affected individuals lack a specific genetic diagnosis. A similar strategy is relevant for other heterogeneous neurological disorders. The affected individuals from different categories; childhood to adult-onset, familial-to-sporadic and pure-to-complex phenotypes can benefit and be diagnosed earlier using modern high throughput sequencing technologies.

## Supporting information

S1 Table List of pathogenic variants identified in eight positive controls. (DOC)

S2 Table Genes included in the HA and HSP gene panel. (DOC)

S3 Table Allele frequencies of the pathogenic and likely-pathogenic variants in local and public databases. (DOC)

S4 Table Diagnostic rate in different categories. (DOC)

S1 Fig Sequencing coverage plot. (DOC)

S1 Appendix Supplementary methods. (DOC)

S2 Appendix Details of clinical features. (DOC)
