# Correction: Targeted high throughput sequencing in hereditary ataxia and spastic paraplegia

**DOI:** 10.1371/journal.pone.0186571

**Published:** 2017-10-12

**Authors:** Zafar Iqbal, Siri L. Rydning, Iselin M. Wedding, Jeanette Koht, Lasse Pihlstrøm, Aina H. Rengmark, Sandra P. Henriksen, Chantal M. E. Tallaksen, Mathias Toft

The last four lines are missing from the legend for Table 3. Please see the complete legend and table here.

**Table 3:** List of variants of uncertain significance.

Chr	Gene	Disorder	OMIM phenotype#	Individual identity	Genomic position (Hg19/GRCH 37)	Transcript	cDNA position	Amino acid position	LOVD variant ID	Zygosity	PhyloP score, in-silico pathogenicity predictions, CADD	Allele frequency in ExAC	Number of affected individuals carrying the variant in the respective family	Main phenotype—additional features
1	*KCND3* ^a^	SCA19 (AD)	607346	HCT-088	g.112322852T>C	NM_004980.4	c.1456A>G	p.(Thr486Ala)	162993	het	4.40/m,p/19.2	0.001461	1	comp AT–pyramidal and extrapyramidal signs
3	*ITPR1*	SCA15/29 (AD)	606658/117360	HCT-029	g.4735396G>A	NM_001168272.1	c.4207G>A	p.(Val1403Met)	162994	het	4.08/s,m,p/17.1	0.00004663	1	pure HSP—none
3	*ITPR1* ^b^	SCA15/29 (AD)	606658/117360	HCT-077	g.4810224G>A	NM_001168272.1	c.5710G>A	p.(Glu1904Lys)	162995	het	3.68/s,m/13.3	0.000008432	1^#^	comp AT—early onset, spastic AT
11	*BSCL2*	SPG17 (AD)	270685	HCT-044	g.62462158C>A	NM_001122955.3	c.512G>T	p.(Arg171Leu)	162996	het	2.14/s,m,p/19.3	0.000008322	1	pure HSP—amyotrophy, neuropathy
11	*SPTBN2*	SCA5 (AD)	600224	HCT-086	g.66453485T>G	NM_00694.2	c.7030A>C	p.(Ser2344Arg)	162997	het	1.66/p/15.1	0.00001679	1^#^	comp AT—neuropathy
11	*SPTBN2*^c^	SCA5 (AD)	600224	HCT-071	g.66453406C>T	NM_00694.2	c.7109G>A	p.(Arg2370His)	162998	het	5.86/s,m,p/33	0.0001252	1^#^	pure AT—none
12	*KIF5A*	SPG10 (AD)	604187	HCT-082	g. 57970109C>T	NM_004984.2	c.2146C>T	p.(Arg716Trp)	162999	het	3.60/s,m,p/24.6	0.00005826	1^#^	comp AT—episodic
15	*TTBK2*	SCA11 (AD)	604432	HCT-115	g.43132604C>G	NM_173500.3	c.245G>C	p.(Gly82Ala)	163000	het	5.21/s,m,p/16	0.0002898	1	comp AT—spastic AT
16	*BEAN1*	SCA31 (AD)	117210	HCT-087	g.66503607T>A	NM_001178020.2	c.128T>A	p.(Ile43Lys)	163001	het	3.35/s,m,p/25.3	-	2	comp AT—lower limb paresis, neuropathy
19	*RTN2*	SPG12 (AD)	604805	HCT-057	g.45996535C>A	NM_005619.3	c.916G>T	p.(Val306Phe)	163002	het	2.71/p/15.6	-	3	pure AT—none

Abbreviations: Chr, chromosome; AD, autosomal dominant; AR, autosomal recessive; OMIM, online Mendelian inheritance in man; cDNA, complementary deoxyribonucleic acid; Zygosity, heterozygous (het), compound heterozygous (c.het), homozygous (hom); LOVD, Leiden open variation database; CADD, combined annotation dependent depletion score, also called as a PHRED score PhyloP, evolutionary conservation score at specific nucleotide position; s, damaging prediction by SIFT (http://sift.jcvi.org); m, damaging prediction by MutationTaster (http://www.mutationtaster.org); p, damaging prediction by PolyPhen-2 (http://genetics.bwh.harvard.edu/pph2/); ExAC, exome aggregation consortium (http://exac.broadinstitute.org); comp, complex; AT, ataxia; HSP, hereditary spastic paraplegia. OMIM gene identifiers: *KCND3* (605411), *ITPR1* (147265), *BSCL2* (606158), *SPTBN2* (604985), *KIF5A* (602821), *TTBK2* (611695).

a Another variant, c.929G>A, p.(Arg310Gln) in *CYP7B1* was found heterozygously in this individual.

b Another variant, c.2261C>T, p.(Pro754Leu) was found in *SPG7* heterozygously in this individual.

c Another variant, c.2228T>C, p.(Ile743Thr) was found in *SPG7* heterozygously in this individual.

#, No additional samples of affected and/or unaffected individuals were available for segregation analysis.
