# Supplementary material for: Targeted high throughput sequencing in hereditary ataxia and spastic paraplegia
Source: PLoS One. 2017 Mar 31;12(3):e0174667. doi: 10.1371/journal.pone.0174667 (PMC5375131; doi:10.1371/journal.pone.0174667)
Supplement: S1 Table — (DOC) [file pone.0174667.s001.doc]

**S1 Table**. List of pathogenic variants identified in eight positive controls

| **Chromosome** | **Gene** | **Transcript** | **cDNA position** | **Amino acid position** | **PHRED/CADD**  **score** | **Zygosity** | **Individual** |
| --- | --- | --- | --- | --- | --- | --- | --- |
| 2 | *SPAST* | NM_014946.3 | c.1378C>T | p.(Arg460Cys) | 21.2 | het | HCT-001 |
| 2 | *REEP1* | NM_001164730.1 | c.3G>A | p.(Met1Ile) | 13.9 | het | HCT-004 |
| 8 | *TTPA* | NM_000370.3 | c.358G>A  c.400C>T | p.(Ala120Thr),  p.(Arg134*) | 19.07  NA | c.het | HCT-013 |
| 9 | *SETX* | NM_015046.5 | c.6792A>G | p.(Ile2264Met | 18.2 | hom | HCT-010 |
| 14 | *ATL1* | NM_015915.4 | c.1259A>C | p.Gln420Pro) | 23.6 | het | HCT-005 |
| 15 | *SPG11* | NM_025137.3 | c.2316+1G>A  c.4162-10T>G | p.?  p.? | NA  NA | c.het | HCT-006 |
| 16 | *SPG7* | NM_003119.2 | c.1454_1462del,  c.2102A>C | p.(Arg485_Glu487del),  p.(His701Pro) | NA  12.8 | c.het | HCT-002 |
| 19 | *PRKCG* | NM_002739.3 | c.417C>A | p.(His139Gln) | 23 | het | HCT-007 |

Abbreviations: cDNA, complementary deoxyribonucleic acid; Zygosity, heterozygous (het), compound heterozygous (c.het), homozygous (hom); CADD, combined annotation dependent depletion score, also called as a PHRED score;. NA, not applicable.
