# Supplementary material for: Targeted high throughput sequencing in hereditary ataxia and spastic paraplegia
Source: PLoS One. 2017 Mar 31;12(3):e0174667. doi: 10.1371/journal.pone.0174667 (PMC5375131; doi:10.1371/journal.pone.0174667)
Supplement: S2 Table — (DOC) [file pone.0174667.s002.doc]

**S2 Table**. Genes included in the HA and HSP gene panel

| | *ABCB7* | | --- | | *ADCK3* | | *AFG3L2* | | *ALS2* | | *ANO10* | | *AP4B1* | | *AP4E1* | | *AP4M1* | | *AP4S1* | | *AP5Z1* | | *APTX* | | *ATL1* | | *ATM* | | *ATN1* | | *ATXN1* | | *ATXN10* | | *ATXN2* | | *ATXN3* | | *ATXN7* | | | *B4GALNT1* | | --- | | *BEAN1* | | *BICD2* | | *BSCL2* | | *c10orf2* | | *CACNA1A* | | *CACNB4* | | *CCDC23* | | *COQ2* | | *CYP2U1* | | *CYP7B1* | | *DARS2* | | *DDHD1* | | *DDHD2* | | *DNMT1* | | *EEF2* | | *ERLIN2* | | *FA2H* | | *FGF14* | | | *FXN* | | --- | | *GBA2* | | *GJB1* | | *GJC2* | | *GRID2* | | *GRM1* | | *HSPD1* | | *ITPR1* | | *KCNA1* | | *KCNA2* | | *KCNC3* | | *KCND3* | | *KCNJ10* | | *KIAA0196* | | *KIAA0226* | | *KIF1A* | | *KIF5A* | | *L1CAM* | | *MARS2* | | | *MRE11A* | | --- | | *NIPA1* | | *PDYN* | | *PLP1* | | *PNPLA6* | | *POLG1* | | *PPP2R2B* | | *PRKCG* | | *REEP1* | | *RTN2* | | *SACS* | | *SETX* | | *SIL1* | | *SLC16A2* | | *SLC1A3* | | *SLC33A1* | | *SPAST* | | *SPG11* | | *SPG20* | | | *SPG21* | | --- | | *SPG7* | | *SPTBN2* | | *SYNE1* | | *TBP* | | *TDP1* | | *TGM6* | | *TPP1* | | *TTBK2* | | *TTPA* | | *VAMP1* | | *VLDLR* | | *VPS37A* | | *ZFYVE26* | | *ZFYVE27* | |
| --- | --- | --- | --- | --- | --- | --- | --- | --- | --- | --- | --- | --- | --- | --- | --- | --- | --- | --- | --- | --- | --- | --- | --- | --- | --- | --- | --- | --- | --- | --- | --- | --- | --- | --- | --- | --- | --- | --- | --- | --- | --- | --- | --- | --- | --- | --- | --- | --- | --- | --- | --- | --- | --- | --- | --- | --- | --- | --- | --- | --- | --- | --- | --- | --- | --- | --- | --- | --- | --- | --- | --- | --- | --- | --- | --- | --- | --- | --- | --- | --- | --- | --- | --- | --- | --- | --- | --- | --- | --- | --- | --- | --- | --- | --- | --- |
