# Supplementary material for: Targeted high throughput sequencing in hereditary ataxia and spastic paraplegia
Source: PLoS One. 2017 Mar 31;12(3):e0174667. doi: 10.1371/journal.pone.0174667 (PMC5375131; doi:10.1371/journal.pone.0174667)
Supplement: S3 Table — (DOC) [file pone.0174667.s003.doc]

**S3 Table. Allele frequencies of the pathogenic and likely-**pathogenic variants in local and public databases

| **Chromosome** | **Gene** | **Genomic position (Hg19)** | **176 in-house exomes** | **230 in-house targeted controls** | **1000g data^a^** | **ESP6500^b^** |
| --- | --- | --- | --- | --- | --- | --- |
| 1 | *KCND3* | g.112329705C>T | - | - | - | - |
| 2 | *SPAST* | g.32341274G>C | - | - | - | - |
| 2 | *KIF1A* | g.241737090T>C | - | - | - | - |
| 2 | *KIF1A* | g.241737148G>A | - | - | - | - |
| 2 | *REEP1* | g.86481833T>C | - | - | - | - |
| 2 | *REEP1* | g.86491145G>A | - | - | - | - |
| 3 | *ITPR1* | g.4776923A>T | - | - | - | - |
| 11 | *BSCL2* | g.62469965C>T | - | - | - | - |
| 11 | *SPTBN2* | g.66472866_66472868del | - | - | - | - |
| 12 | *KIF5A* | g.57962782G>A | - | - | - | - |
| 13 | *SACS* | g.23905327G>A | - | - | - | - |
| 13 | *SACS* | g.23905354G>C | - | - | - | - |
| 14 | *ATL1* | g.51088610T>C | - | - | - | - |
| 16 | *SPG7* | g.89576947T>A | - | - | - | - |
| 16 | *SPG7* | g.89613145C>T  g.89616910A>T | 0.005  - | 0.004  - | 0.0014  - | 0.003463  0.000077 |
| 16 | *SPG7* | g.89613145C>T  g.89620367A>C | 0.005  - | 0.004  - | 0.0014  - | 0.003463  - |
| 16 | *SPG7* | g.89613145C>T  g.89620367A>C | 0.005  - | 0.004  - | 0.0014  - | 0.003463  - |
| 18 | *AFG3L2* | g.12337401T>C | - | - | - | - |
| 19 | *CACNA1A* | g.13323200C>T | - | - | - | - |
| 19 | *PRKCG* | g.54393142T>C | - | - | - | - |
| 20 | *TGM6* | g.2384113G>A | - | - | - | - |

^a^1000 genomes data (1000g), http://www.1000genomes.org. ^b^Exome sequencing project (ESP6500), http://evs.gs.washington.edu/EVS/.
