# Supplementary material for: Targeted high throughput sequencing in hereditary ataxia and spastic paraplegia
Source: PLoS One. 2017 Mar 31;12(3):e0174667. doi: 10.1371/journal.pone.0174667 (PMC5375131; doi:10.1371/journal.pone.0174667)
Supplement: S4 Table — (DOC) [file pone.0174667.s004.doc]

**S4 Table. Diagnostic rate in different categories**

|  | **Percentage with pathogenic and likely-pathogenic variants** | **Autosomal dominant (AD) (%)** | **Autosomal recessive (AR) (%)** | **Sporadic (SPO) (%)** | **Consanguinity (%)** | **Pure (%)** | **Complex (%)** |  |
| --- | --- | --- | --- | --- | --- | --- | --- | --- |
| **HA** | 14 | 14 | 10 | 17 | 25 | 11 | 17 |  |
| **HSP** | 26 | 15 | 36 | 20 | 33 | 32 | 21 |  |
| **Total** | 19 | 15 | 24 | 19 | 29 | 19 | 19 |  |
