# Supplementary material for: Targeted high throughput sequencing in hereditary ataxia and spastic paraplegia
Source: PLoS One. 2017 Mar 31;12(3):e0174667. doi: 10.1371/journal.pone.0174667 (PMC5375131; doi:10.1371/journal.pone.0174667)
Supplement: S1 Fig — (DOC) [file pone.0174667.s005.doc]

**S1 Fig. Sequencing coverage plot**

**
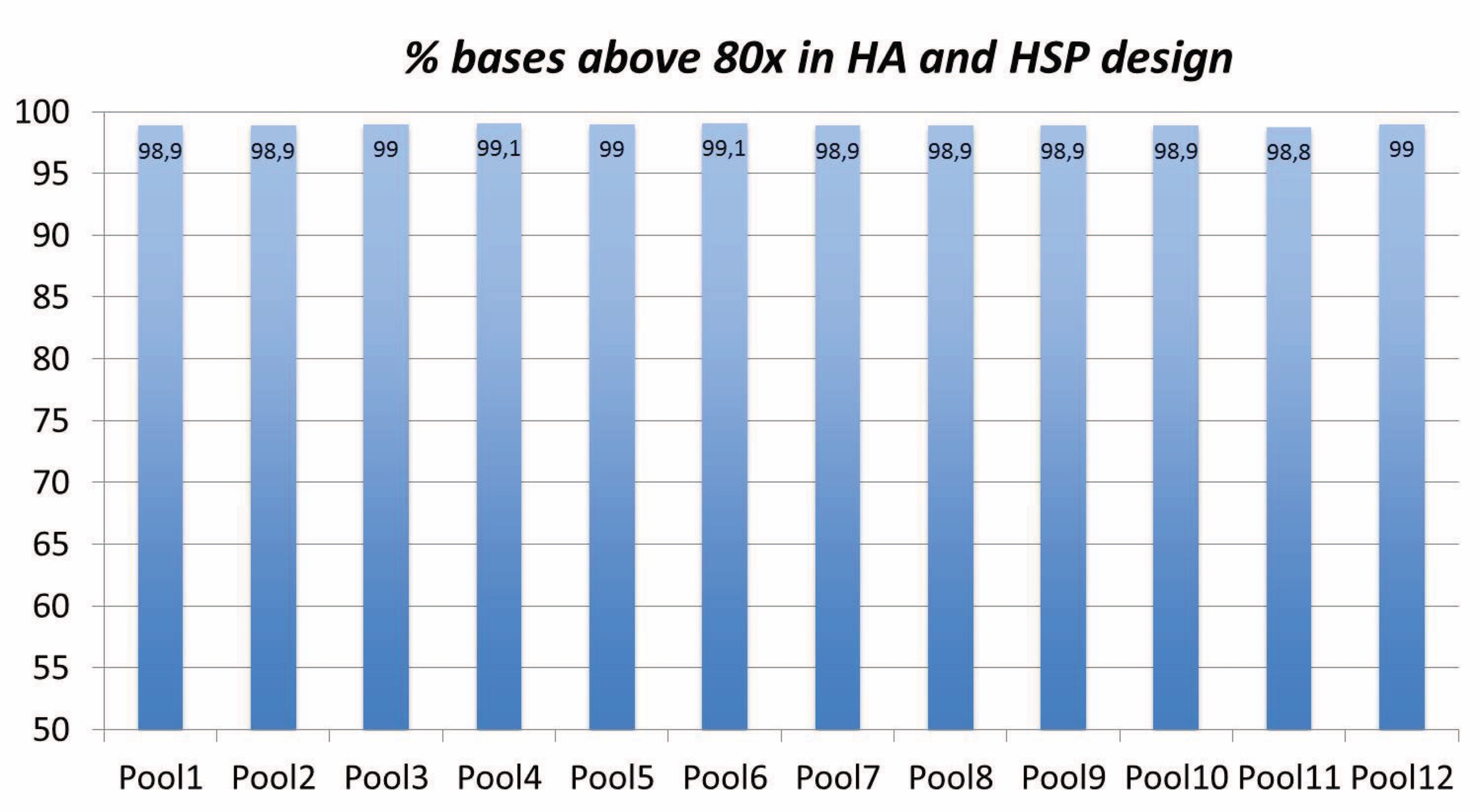
**

A graph which explains the proportion of target exonic positions covered above 80x across all 12 pools.
