# Supplementary material for: Targeted high throughput sequencing in hereditary ataxia and spastic paraplegia
Source: PLoS One. 2017 Mar 31;12(3):e0174667. doi: 10.1371/journal.pone.0174667 (PMC5375131; doi:10.1371/journal.pone.0174667)
Supplement: S1 Appendix — (DOC) [file pone.0174667.s006.doc]

**S1 Appendix**

**Supplementary Methods**

**Sanger Sequencing**

Primers for amplification of the exons carrying variants were designed by the Primer3 program (http://bioinfo.ut.ee/primer3-0.4.0/). Primer sequences are available on request. PCR reactions were carried out on 40ng of genomic DNA with Dream*Taq* PCR mix (Life Technologies, Carlsbad, CA). PCR products were purified by using ExoSAP-IT (Affymetrix, Santa Clara, CA), according to the manufacturer’s protocol. PCR amplicons were sequenced by external provider GATC BIOTECH (Konstanz, Germany). To align the sequences and detect expected variants, Sequencher 5.1v (Gene Codes Corporation, Ann Arbor, MI) was used.
