# Supplementary material for: Targeted high throughput sequencing in hereditary ataxia and spastic paraplegia
Source: PLoS One. 2017 Mar 31;12(3):e0174667. doi: 10.1371/journal.pone.0174667 (PMC5375131; doi:10.1371/journal.pone.0174667)
Supplement: S2 Appendix — (DOC) [file pone.0174667.s007.doc]

**S2 Appendix**

**Clinical Features of probands with pathogenic and likely-pathogenic variants**

*AFG3L2* (SCA 28, MIM 610246)

The proband HCT-067 had a juvenile-onset, slowly progressive gait and limb ataxia, inherited in a possible autosomal dominant pattern with an affected sister and father. At the age of 48 years, she scored 5/40 on the Scale for the Assessment and Rating of Ataxia (SARA) score. She had mild gait and limb ataxia, mild dysarthria and dysphagia. No spasticity was found. Reflexes were decreased in the lower limbs. In addition, there was saccadic pursuit and slow saccades when tested for eye motility, but no ptosis.

*BSCL2* (SPG17, MIM 270685)

The proband, HCT-051 was a man aged 27 at inclusion in the study. His maternal grandfather was of Asian origin, but there were to the best of his knowledge no similar cases in the family. He had two healthy siblings. Onset of symptoms was in his teens, when the gym teacher noticed a stiff gait due to shortening of the achilles tendons, which were subsequently operated with good results. At examination he had bilateral distal motor deficit and spastic gait. There was in addition motor deficit in hands, hyperreflexia and inverted plantar response bilaterally. Axonal motor neuropathy was confirmed by neurography.

The description fits well with earlier reported phenotypes of SPG17. It is worth mentioning that distal amyotrophy in patients with HSP remains very rare. This should prompt a particular interest for SPG17 in those patients and encourage genetic testing.

*CACNA1A* (EA2, MIM 108500)

The proband HCT-059 had an early onset episodic ataxia which was untreated until he was diagnosed at the age of 38. From early childhood until he received treatment he had 3-4 weekly attacks lasting hours to days. Attacks were triggered by stress or alcohol and associated with dizziness, vomiting, unsteadiness, anxiety and dysarthria. No other symptoms, particularly no migraine, epilepsy, nor hemiplegia were observed during attacks. At inclusion in the study at the age of 42 years, he scored 2/40 on the SARA scale with mild gait ataxia and problems standing with feet together. Nystagmus and saccadic pursuit were observed when tested for slow pursuit. After treatment with [acetazolamide](http://www.drugs.com/mtm/acetazolamide.html), the attacks were less frequent, milder and shorter. The disorder was transmitted in an autosomal manner in this family. No other family members were available for segregation analysis.

*ITPR1* (SCA15/29, MIM 606658/117360)

The proband HCT-080 had onset of symptoms at the age of 10 years, and severe disability at examination after 41 years’ disease duration. We have examined four affected family members in this family, with a range of age of onset of unsteadiness from 4 to 35 (4, 7, 10, 35) years. The clinical symptoms of all the affected in the family were a slowly progressive ataxia with polyneuropathy, without tremor or any cognitive impairment. Clinically, this could fit the descriptions of SCA15. Due to the uncertainty on phenotypic outcomes of *ITPR1*-disorder, we have designated the phenotype as SCA15/SCA29 in this family.

*KIF5A* (SPG10, MIM 604187)

A known pathogenic mutation was found in *KIF5A* in HCT-043. This proband was clinically classified as a complex spastic paraplegia with cognitive impairment and axonal sensorimotor polyneuropathy. Other family members had a pure spastic paraplegia with mild sensory involvement, typical of the SPG10 phenotype. Interestingly, array CGH analysis has showed a 22q11.21 duplication in the proband, also with a dominant inheritance in the family, but not segregating with the spastic paraplegia phenotype. This illustrates that the combination of apparently different phenotypes of a single genotype in a family, may result from the co-existence of two different disorders.

*PRKCG* (SCA14, MIM 605361)

The proband HCT-118 had slowly progressive gait and limb ataxia from the age of 23 years. At the age of 48 years she still walked unaided, despite gait ataxia and spasticity. Her upper and lower limb reflexes were increased, but with normal plantar responses. She had mild vibration sense deficiency, but no other sensory findings. Mild dystonia was present in the feet; there was hypomimia, slight dysphagia and saccadic pursuit when tested for slow pursuit. SARA score was 12.5/40. The disorder was transmitted in a possible autosomal dominant manner in this family, with a deceased mother who had the same symptoms. MRI of the brain was done when she was 48 years old and showed atrophy of the cerebellum.

*SPG7* (SPG7, MIM 607259)

SPG7 represents a heterogenous phenotype ranging from complex spastic paraplegia with ataxia and ophthalmoplegia to a pure cerebellar ataxia. Probands HCT-033, HCT-112 and HCT-116 had a core phenotype of spastic ataxia with ophthalmoplegia and bladder disturbances (Rydning SL *et al*, Eur J Neurol, 2016). Proband HCT-048 was of non-caucasian origin, and developed spastic paraplegia from 40 years of age, a complex phenotype combined with mild cerebellar ataxia, saccadic pursuit, nystagmus and mild sensory deficits, and unlike the Norwegian patients this proband did not have ptosis. At 45 years of age brain MRI showed mild cerebellar atrophy and EMG/neurography was normal. A sibling had similar gait disturbances, but no other family members were available for segregation analysis.

*TGM6* (SCA35, MIM 613908)

A novel variant was identified in *TGM6* in the proband HCT-101. The proband experienced clumsiness from 10 years of age. Upon examination at age 55, this proband had cerebellar ataxia, postural tremor and dystonia. There were also pyramidal signs comprising mild spasticity and inverted plantar response, and signs of posterior column dysfunction. The SARA score was 10. MRI did not show evident cerebellar atrophy. There was no other known affected in the family history and no other family members were available for segregation analysis. The phenotype fits with SCA35, which is typically described as a slowly progressive cerebellar ataxia with pyramidal signs and tremor, without cognitive impairment.

**Phenotypic description on carriers of variants of uncertain significance**

The proband with the *KCND3* (SCA19, MIM 607346) variant presented with an episodic ataxia-like phenotype, short episodes of dizziness and stiffness from early childhood. The proband, HCT-044 with the *BSCL2* (SPG17, MIM 270685) variant was a woman aged 57 at inclusion in the study and presented with pure HSP phenotype, amyotrophy and neuropathy. EMG and neurography findings were consistent with an axonal motor neuropathy. The proband had no knowledge of similar symptoms in the family. Both children of the proband were healthy. The proband HCT-077 with an *ITPR1* (SCA15, MIM 606658 and SCA29, MIM 117360) variant presented with a somewhat atypical HSP picture from the age of 60 years old. Although heterogeneity is reported in SCA15 and SCA29, the phenotype is not consistent with previous reports. The proband HCT-077 with *ITPR1* variant had an early onset complex ataxia with dysarthria, cognitive impairment, rigidity, spasticity, paralysis in lower limbs, which could be compatible with SCA29. Both probands with a variant in the *SPTBN2* gene had a phenotype consistent with previous reports of SCA5 (MIM 600224). The proband with a *KIF5A* variant presented with an episodic ataxia-like phenotype, from the age of ten years. Her phenotype is atypical for SPG10 (MIM 604187), but cannot be excluded. The proband with the variant in *TTBK2* presented with benign slow progressive spastic ataxia such as previously reported for SCA11 (MIM 604432). The proband with a variant in *BEAN1* (SCA31, MIM 117210) presented with a reasonable concordance of the phenotype with the reported phenotype. A novel variant in *RTN2* was identified in HCT-057 and segregated with the phenotype in three affected individuals. The proband with a *RTN2* variant had a pure gait ataxia from the age of 50 years. This phenotype fits poorly with previously reported SPG12 (MIM 604805).
